# Systemic inflammasome activation and pyroptosis associate with the progression of amnestic mild cognitive impairment and Alzheimer’s disease

**DOI:** 10.1186/s12974-021-02329-2

**Published:** 2021-12-02

**Authors:** Wenjuan Rui, Hong Xiao, Yi Fan, Zhongxuan Ma, Ming Xiao, Sheng Li, Jingping Shi

**Affiliations:** 1 Department of Neurology, Affiliated Brain Hospital of Nanjing Medical University, 264 Guangzhou Road, Nanjing, 210029 Jiangsu China; 2 Department of the Neuro-Psychiatric Institute, Affiliated Brain Hospital of Nanjing Medical University, 264 Guangzhou Road, Nanjing, 210029 Jiangsu China; 3 Department of Pharmacy, Affiliated Brain Hospital of Nanjing Medical University, 264 Guangzhou Road, Nanjing, 210029 Jiangsu China; 4 Jiangsu Key Laboratory of Neurodegeneration, Department of Pharmacology, Nanjing Medical University, 101 Longmian Avenue, Nanjing, 211166 Jiangsu China

**Keywords:** Alzheimer’s disease, Gasdermin D, Neuroinflammation, Inflammasome, Pyroptosis, Interleukin-1β

## Abstract

**Background:**

Growing evidence indicates that inflammasome-mediated inflammation plays important roles in the pathophysiology of amnestic mild cognitive impairment (aMCI) and Alzheimer’s disease (AD). Pyroptosis induced by inflammasome, and Gasdermin D (GSDMD) is involved in several neurodegenerative disorders. However, it is not clear whether peripheral inflammasome and pyroptosis are activated in aMCI and AD patients, influencing on neuroinflammation. The aim of this study was to examine the association between systemic inflammasome-induced pyroptosis and clinical features in aMCI and AD.

**Methods:**

A total of 86 participants, including 33 subjects with aMCI, 33 subjects with AD, and 20 cognitively normal controls, in this study. The Mini Mental State Examination (MMSE) and the Montreal Cognitive Assessment (MoCA) scale were used for cognitive assessment. Levels of inflammasome-related genes/proteins in peripheral blood mononuclear cells (PBMCs) were determined using quantitative polymerase chain reaction and Western blotting. The levels of IL-1β, Aβ1-42, Aβ1-40, p-tau, and t-tau in cerebrospinal fluid (CSF), as well as the plasma IL-1β level, were measured by enzyme-linked immunosorbent assay. Finally, lipopolysaccharides (LPS) were used to investigate the effects of systemic inflammasome-induced pyroptosis in an AD mice model.

**Results:**

Several genes involved in the inflammatory response were enriched in PBMCs of AD patients. The mRNA and protein levels of NLRP3, caspase-1, GSDMD, and IL-1β were increased in PBMCs of aMCI and AD patients. The IL-1β level in plasma and CSF of aMCI and AD patients was significantly higher than that in controls and negatively correlated with the CSF Aβ1-42 level, as well as MMSE and MoCA scores. Furthermore, there was a positive correlation between the IL-1β level in plasma and CSF of aMCI or AD patients. In vivo experiments showed that systemic inflammasome-induced pyroptosis aggravated neuroinflammation in 5 × FAD mice.

**Conclusions:**

Our findings showed that canonical inflammasome signaling and GSDMD-induced pyroptosis were activated in PBMCs of aMCI and AD patients. In addition, the proinflammatory cytokine IL-1β was strongly associated with the pathophysiology of aMCI and AD. As such, targeting inflammasome-induced pyroptosis may be a new approach to inhibit neuroinflammation in aMCI and AD patients.

**Supplementary Information:**

The online version contains supplementary material available at 10.1186/s12974-021-02329-2.

## Background

Alzheimer’s disease (AD) is the most common neurodegenerative disease. Beta-amyloid (Aβ) deposition and neurofibrillary tangle formation in the brain parenchyma, which cause neuronal death and cognitive decline, are believed to be the main features of the disease [1]. Although many pharmaceutical companies have developed drugs to alleviate the symptoms of Aβ accumulation, most of them have failed in clinical settings [2, 3]. Recently, although the United States Food and Drug Administration approved the Aβ monoclonal antibody aducanumab, which was developed by Biogen Co., to treat AD [4], there is much controversy on the use of this drug and its long-term effects need to be further verified. Therefore, it is important to completely understand the pathogenesis of AD.

Neuroinflammation plays important roles in several neurodegenerative diseases, such as Parkinson’s disease, amyotrophic lateral sclerosis, amnestic mild cognitive impairment (aMCI), and AD [5–8]. Studies have reported that chronic neuroinflammation can occur prior to Aβ and tau pathologies in AD [9]. Proinflammatory cytokines, such as interleukin (IL)-1β, can drive neuroinflammation, and their levels are upregulated in the brain and cerebrospinal fluid (CSF) of AD patients [10]. Moreover, proinflammatory cytokines can penetrate the blood–brain barrier (BBB), thereby promoting inflammation in the brain [11]. Goehler et al. demonstrated that intraperitoneal injection of IL-1β can induce inflammation in the brain, indicating that this cytokine can mediate crosstalk between the immune system and the central nervous system (CNS) [12]. Other studies have reported an association between the plasma IL-1β level and the progression of aMCI and AD [13, 14]. However, there is no study on the association between IL-1β level in CSF and inflammation activation in peripheral blood mononuclear cells (PBMCs) of aMCI and AD patients.

Inflammasomes are cytosolic multi-protein complexes that recognize many stimulatory signals and trigger inflammatory cytokine production [15]. Upon recognition of stimuli from pathogens and injured cells, the levels of inflammasome-associated pattern recognition receptors (PRRs), such as NLRP1, NLRP3, and NLRC4, which are members of the NOD-like receptor (NLR) family, as well as absent-in-melanoma 2 (AIM2) and PYRIN-CARD protein ASC, increased, resulting in the oligomerization of cytosolic protein complexes [16]. These events activated caspase signaling and induced IL-1β and IL-18 production [15]. Increasing evidence indicates that the induction of proinflammatory cytokines, such as IL-1β, contributes to neuroinflammation in AD patients [17]. Pyroptosis, which is different from apoptosis and necroptosis, is a type of programmed cell death characterized by DNA fragmentation, cellular swelling, and membrane rupture that is triggered by inflammasomes [18]. Gasdermin D (GSDMD), a substrate of caspases-1 and -4/11, has been reported to be the executioner of pyroptosis. GSDMD cleavage results in N-terminal fragment oligomerization and plasma membrane pore formation, which regulate the secretion of proinflammatory cytokines, including IL-1β [18]. Studies have reported that GSDMD-induced pyroptosis is involved in several neurological disorders, such as ischemic stroke [19], Parkinson’s disease [20], and multiple sclerosis [21]. However, it is unclear whether GSDMD can induce pyroptosis in periphery of aMCI and AD patients and aggravate neuroinflammation.

The aim of this study was to investigate the association between systemic inflammasome-mediated pyroptosis and aMCI or AD progression.

## Materials and methods

### Participants

We used the criteria of the National Institute on Aging and the Alzheimer’s Association for AD diagnosis [22], and published criteria [23] for aMCI diagnosis. We recruited 33 patients with aMCI and 33 patients with AD from the Department of Neurology, Affiliated Brain Hospital of Nanjing Medical University, from January 2019 to April 2020. For controls, we recruited 20 age-matched subjects with (i) no history of infectious, inflammatory, and autoimmune diseases; (ii) no history of psychiatric and memory disorders; and (iii) no history of prescription and non-prescription drug use. All participants or their legal guardians provided informed written consent. This study was approved by the Institutional Review Board of the Affiliated Brain Hospital of Nanjing Medical University.

### Collection of peripheral blood mononuclear cells

Blood was collected in the morning and processed within 4 h. Approximately 3 mL of anti-coagulated whole blood was centrifuged at 3500 rpm for 3 min at 4 °C, and plasma was collected and stored at − 80 °C for the measurement of cytokine levels. To obtain PBMCs, 2 mL of whole blood was diluted with phosphate-buffered saline (PBS, 1:1) and transferred to centrifuge tubes containing 3 mL of Ficoll Paque (GE Healthcare, Uppsala, Sweden). After centrifugation at 400×*g* for 20 min at room temperature, PBMCs were collected and washed twice in 10 mL of PBS. Cells were re-suspended in TRIzol reagent or lysis buffer for the isolation of RNA or protein, respectively.

### Quantitative polymerase chain reaction analysis

Total RNA extraction, cDNA synthesis, and quantitative polymerase chain reactions (qPCR) were performed according to the manufacturer’s instructions. TRIzol reagent (Invitrogen, Waltham, MA, USA) was used to extract RNA from PBMCs, and RNA was used for cDNA synthesis. qPCR was performed using SYBR Premix Ex Taq (Vazyme, Nanjing, China). The following primers were used: *Il-1b* (5′-TGTAGTGGTGGTCGGAGATT-3′, forward; 5′-ATGATGGCTTATTACAGTGGC-3′, reverse), *Nlrp3* (5′-AGGGCGTTGTCACTCAGGT-3′, forward; 5′-TCGGAGATTGTGGTTGGG-3′, reverse), *Gsdmd* (5′-AGTGCCAGGGAGGCGTAGAGT-3′, forward; 5′-TGGGTCTTGCTGGACGAGTG-3′, reverse), *Casp1* (5′-GGAAGAGCAGAAAGCGATAA-3′, forward; 5′-TTGAAGGACAAACCGAAGG-3′, reverse), *Casp4* (5′-TGCCAGGAAAGAGGTAGAAA-3′, forward; 5′-TCGGAAGGTACAGCAATCA-3′, reverse), *Nlrc4* (5′-GACTAATGCTGGATCAGGTAG-3′, forward; 5′-TTTGGCGGGAAATCGTGT-3′, reverse), *Aim2* (5′-TCAGTACCATAACTGGCAAA-3′, forward; 5′-AGAAATGATGTCGCAAAGC-3′, reverse), *Nlrp1* (5′-AACGTAGAACTCCGAGAAC-3′, forward; 5′-CGAATCCACAAGCCACCC-3′, reverse), and *Gapdh* (5′-GAAGGTGAAGGTCGGAGTC-3′, forward; 5′-GAAGATGGTGATGGGATTTC-3′, reverse). The relative expression level of each target gene was calculated using a standard curve and normalized against the *Gapdh* expression level.

### Immunofluorescence staining

PBMCs were collected, fixed with 4% paraformaldehyde in 1.5-mL centrifuge tubes for 30 min at 4 °C, washed twice with PBS, blocked with 3% goat serum, and incubated with a rabbit anti-cleaved N-terminal GSDMD primary antibody (1:50, Abcam, Cambridge, UK) that as diluted with PBS containing 3% serum and 2 mM EDTA for 12 h at 4 °C, followed by incubation with an Alexa Fluor 555 anti-rabbit secondary antibody (1:500, Invitrogen). For in vivo experiments, mouse brains were fixed with 4% paraformaldehyde, dehydrated with 30% sucrose, embedded in optimal cutting temperature compound, and sectioned at 25-μm thickness. Tissue sections were blocked, incubated with a rabbit anti-IBA1 (1:500, Wako, Tokyo, Japan), mouse anti-GFAP (1:500, Sigma, St. Louis, MA, USA), or mouse anti-NeuN (1:200, Millipore, Burlington, CA, USA) primary antibody, followed by incubation with an Alexa Fluor 488 anti-rabbit (1:500, Invitrogen) or Alexa Fluor 555 anti-mouse (1:500, Invitrogen) secondary antibody and treatment with 4′,6-diamidino-2-phenylindole (DAPI) or thioflavin S (Sigma-Aldrich).

### Collection of cerebrospinal fluid

CSF was collected by lumbar puncture at the L3/L4 or L4/L5 level in the morning. The first 20 drops of CSF were discarded and then approximately 2 mL of CSF was collected in a polypropylene tube. Specimens were centrifuged at 2000×*g* for 10 min at room temperature to eliminate cells and other insoluble materials, aliquoted, and stored at − 80 °C for further processing.

### Enzyme-linked immunosorbent assay

Plasma and CSF specimens were used to measure the protein levels of AD biomarkers and IL-1β. The levels of AD biomarkers, including Aβ1-40, Aβ1-42, p-tau-181, and t-tau, were measured with commercially available enzyme-linked immunosorbent assay (ELISA) kits (INNOTEST, Fujirebio, Ghent, Belgium) according to the manufacturer’s instructions. Likewise, the IL-1β level in plasma and CSF was measured with a commercial ELISA kit (Jingmei, Yancheng, China). A microplate reader (Antobio, Zhengzhou, China) was used to obtain absorbance readings. For in vivo experiments, blood and brain specimens were used to measure the IL-1β protein level with a commercial ELISA kit (Abcam, Cambridge, UK).

### Western blot analysis

Approximately 1.5 × 10^6^ PBMCs were combined with 70 μL of loading buffer (Yeasen, Shanghai, China) and heated for 10 min at 99 °C. Equivalent amounts of protein were loaded onto SDS–polyacrylamide gels and then blotted onto PVDF membranes. Membranes were incubated overnight at 4 °C with a mouse anti-NLRP3 (1:1000, AdipoGen Corp., San Diego, CA, USA), rabbit anti-caspase-1 (1:1000, Abcam, Cambridge, UK), rabbit anti-GSDMD (1:200, Novus, Centennial, CO, USA), mouse anti-IL-1β (1:1000, R&D Systems, Minneapolis, MN, USA), or mouse anti-β-actin (1:4000, Sigma-Aldrich) primary antibody. Membranes were washed three times with Tris-buffered saline and incubated with an IRDye 800CW goat-anti-mouse/rabbit secondary antibody (1:3000, LI-COR Biosciences, Lincoln, NE, USA). Image J software was used to analyze the intensity of target protein bands. For in vivo experiments, mouse spleens were harvested and homogenized in homogenization buffer (20 mM Tris–HCl, 150 mM NaCl, 1 mM EGTA, 1 mM EDTA, 2.5 mM sodium pyrophosphate, 1 mM β-glycerol phosphate, 1 mM sodium vanadate, 1% Triton X-100, 1 mg/mL leupeptin, and protease inhibitor cocktail, pH 7.5) at a tissue:buffer ratio of 1:10. The concentration of each protein sample was quantified using the bicinchoninic acid method after centrifuging at 12,000×*g* for 10 min. Protein samples were subjected to Western blot analysis as described above.

### RNA-sequencing analysis

For RNA-sequencing (RNA-seq) analysis, PBMCs were obtained. RNA isolation, cDNA library construction, and RNA-seq were performed using the BGISEQ-500 system (Beijing Genomic Institution, Beijing, China). Clean reads were mapped to the human genome (hg38) by HISAT2, and matched reads were calculated and normalized to FPKM. Fold changes were calculated for all possible comparisons. To select genes with significant changes in expression, a 1.5-fold cutoff was applied. Gene Ontology Biological Process (GO-BP) analysis was performed using R package. Target genes were filtered using significantly different gene expression (*P* < 0.05). The sequencing data have been deposited in the NCBI Sequence Read Archive (SRA) database under the accession code PRJNA726043.

### Treatment of mice with lipopolysaccharides

We used 5-month-old female 5 × FAD mice and littermate wild-type (WT) mice that were kindly provided by Dr. Ming Xiao (Nanjing Medical University, Jiangsu, China). These mice carried mutations in both human amyloid precursor protein (APP695) and presenilin-1 (PSEN1) genes. The APP gene contained three familial AD (FAD) mutations, namely, Swedish (K670N, M671L), Florida (I716V), and London (V717I). The PSEN1 gene contained two FAD mutations, namely, M146L and L286V. Lipopolysaccharides (LPS; 500 μg/kg) or PBS was intraperitoneally injected 48 h before sacrificing the mice. For GSDMD inhibitor treatment experiments, disulfiram (50 mg/kg) or PBS was intraperitoneally injected 4 h before challenging with LPS. Blood, as well as brain and spleen specimens, were collected for ELISA, Western blot analysis, and immunofluorescence staining (*n* = 3/group). Animal experiments were conducted according to the National Institutes of Health Guidelines for the Care and Use of Laboratory Animals, and all animal procedures were approved by the Ethical Review Committee for Laboratory Animal Welfare of Nanjing Medical University.

### Statistical analysis

Data are presented as the mean ± standard error of the mean (SEM). Statistical analysis was performed with one-way ANOVA, followed by Sidak’s multiple comparisons test or unpaired *t* test. Correlation analysis was performed using a linear regression model. Chi-square test was used for the analysis of discrete variables, such as gender. Statistical analysis was performed with GraphPad Prism 6.0 software (Graph Pad Software Inc., San Diego, CA, USA). *P* values were indicated as *P < 0.05, **P < 0.01, and ***P < 0.001.

## Results

### Participant characteristics

A total of 66 patients and 20 controls were enrolled in this study. Participant characteristics are listed in Table 1. No statistical differences were found in age or gender between aMCI, AD, and controls groups. Mini-Mental State Examination (MMSE) and Montreal Cognitive Assessment (MoCA) scores were significantly different between aMCI and controls groups, aMCI and AD groups, and AD and controls groups. The expression of AD biomarkers, including Aβ1-42, Aβ1-40, and p-tau-181, in CSF differed between aMCI patients and AD patients.

**Table 1:** Clinical characteristics of study participants

Variables	Control (*n* = 20)	aMCI (*n* = 33)	AD patients (*n* = 33)
Gender (female/male)	11/9	23/10	23/10
Age (years)	59.90 ± 6.18	63.61 ± 8.88	65.66 ± 9.22
MMSE	29.13 ± 1.12	18.19 ± 1.19^####^	12.52 ± 1.07***^####^
MoCA	29.22 ± 1.38	12.79 ± 1.15^####^	7.03 ± 0.84***^####^
CSF level (Aβ 1-42) pg/mL	N.A.	628.00 ± 64.71	446.60 ± 29.95*
CSF level (Aβ 1-40) pg/mL	N.A.	11,410.00 ± 431.50	9842.00 ± 453.90*
CSF level (Aβ 1-42/Aβ 1-40)	N.A.	0.056 ± 0.0058	0.045 ± 0.0031
CSF level (p-tau-181) pg/mL	N.A.	90.85 ± 10.12	138.10 ± 19.88*
CSF level (t-tau) pg/mL	N.A.	416.50 ± 26.76	411.60 ± 33.80

Numbers are expressed as mean ± SEM

*N.A.* not available

*P < 0.05, ***P < 0.001 versus aMCI group, ^####^*P* < 0.001 versus control group

### Inflammation-related genes and signaling pathways are enriched in PBMCs of AD patients

**Fig. 1:**
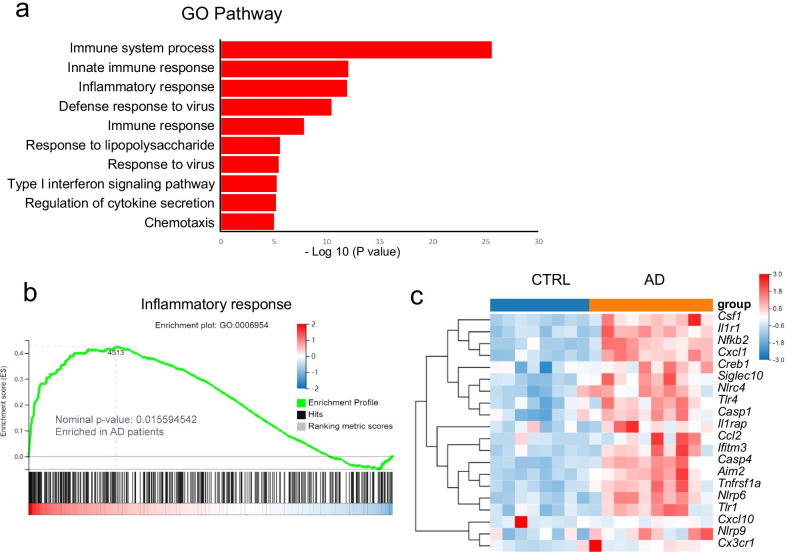
RNA-seq analysis of PBMCs isolated from patients with AD and controls. **a** GO-BP analysis showing the most significantly enriched signaling pathways in PBMCs. **b** GSEA of the genes associated with the “inflammatory response” in PBMCs based on the Gene Ontology Biological Process Database. Nominal *P* < 0.05. **c** Heatmap of genes with an adjusted *P* value < 0.05, false discovery rate < 0.05, and log_2_ fold-change > 1.5 from RNA-seq analysis of PBMCs. *n* = 8 for control, *n* = 10 for AD

Inflammasomes have important roles in the progression of neuroinflammation. To investigate whether a relationship exists between peripheral inflammasome activation and dementia, we first investigated changes in the gene transcriptome of PBMCs isolated from AD and controls by performing RNA-seq analysis. GO biological process analysis indicated that the most significantly changed gene enrichment pathways in AD patients were immune system processes, innate immune system responses, and inflammatory responses (Fig. 1a). Moreover, gene network and gene set enrichment analysis (GSEA) revealed enrichment in the up-regulated genes involved in the inflammatory response pathway in AD patients (Fig. 1b). These findings agreed with those of the heatmap, which showed that the levels of many genes involved in inflammasome-mediated inflammation, such as *Il1r1*, *Il1rap*, *Nlrc4*, *Nlrp6*, *Nlrp9*, *Aim2*, *Casp1*, and *Casp4*, were increased in PBMCs of AD patients (Fig. 1c). As such, these genomic analyses indicate that inflammasome-mediated inflammation pathways are up-regulated in AD patients.

### Inflammasome activation and GSDMD expression in PBMCs of AD and aMCI patients

**Fig. 2:**
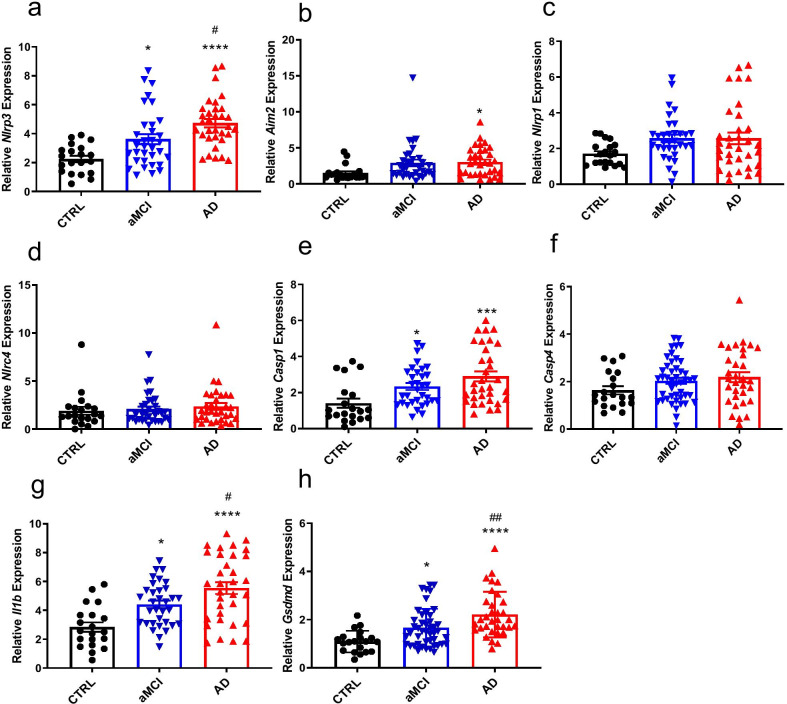
Expression of inflammasome-related genes in PBMCs isolated from patients with AD and controls. **a**–**h** Expression levels of the indicated genes relative to GAPDH as determined by RT-qPCR analysis. *P < 0.05, ***P < 0.001, ****P < 0.001 versus control group, ^#^*P* < 0.05, ^##^*P* < 0.01 versus aMCI group. *n* = 20 for control, *n* = 33 for aMCI, *n* = 33 for AD. Data are expressed as means ± SEM. One-way ANOVA

To further confirm the changes in inflammasomes, we increased the number of clinical samples for the analysis of the expression of inflammasome-related genes by qPCR. As shown in Fig. 1, NLRP3 and AIM2 mRNA levels in patients with aMCI or AD were higher than those in controls (Fig. 2a, b), but NLRP1 and NLRC4 expression did not differ among the groups (Fig. 2c, d). Next, we examined the expression of downstream effector proteins. Caspase-1 and IL-1β mRNA levels in patients with aMCI or AD were higher than those in controls, but caspase-4 expression did not differ among the groups (Fig. 2e–g). These data were partially consistent with our genomic results. GSDMD has been extensively applied in studies of pyroptosis, and the release of IL-1β is highly dependent on the activation of GSDMD [24, 25]. Therefore, we measured the GSDMD level in patients with aMCI or AD, and to the best of our knowledge, this is the first study to examine GSDMD expression in these populations. Compared with the control group, the GSDMD mRNA level in patients with aMCI or AD was higher than that in controls, as expected (Fig. 2h).

### Inflammasome-induced pyroptosis drives systemic inflammation in patients with aMCI and AD

**Fig. 3:**
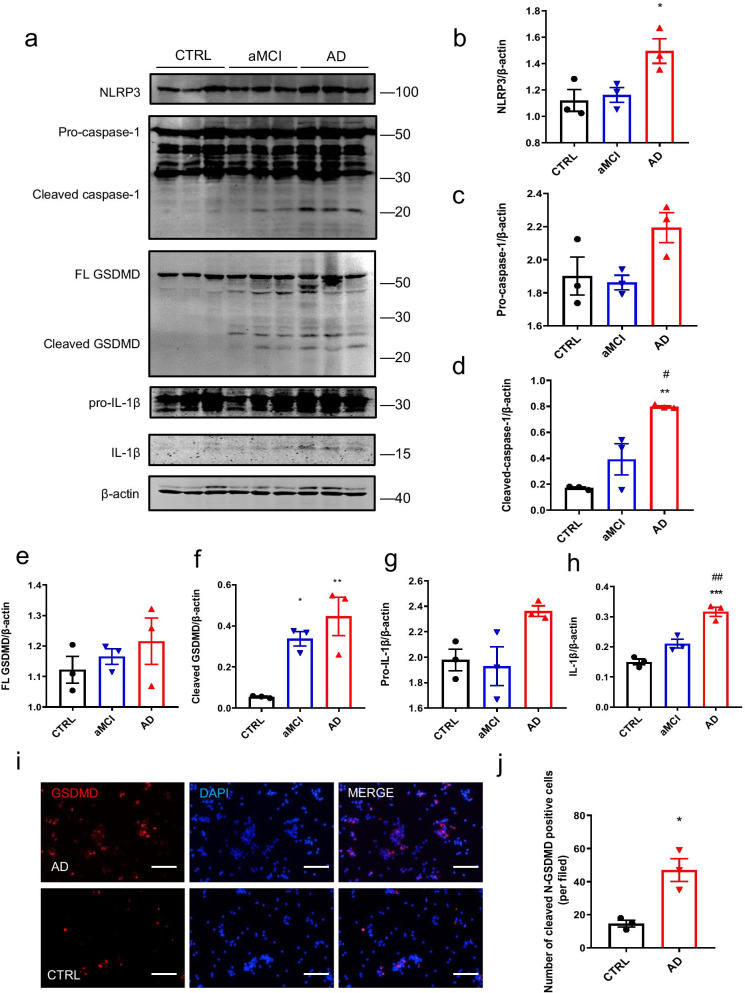
Activation of proteins related to inflammasomes and pyroptosis in PBMCs isolated from patients with AD and controls. **a** Expression and activation of NLRP3, caspase-1, GSDMD, and IL-1β in PBMCs as determined by Western blot analysis. **b**–**h** Quantification of relative protein levels shown in **a**. **i** Immunofluorescence analysis of GSDMD (N-terminus) in PBMCs isolated from a representative AD patient and control. **j** Quantification of positive cells shown in **i**. *P < 0.05, **P < 0.01, ***P < 0.001 versus control group, ^#^*P* < 0.05, ^##^*P* < 0.01 versus aMCI group. Data are expressed as means ± SEM (*n* = 3). One-way ANOVA for **b**–**h**. Unpaired *t* test for **j**

To confirm the biochemical changes in inflammasomes of PBMCs isolated from aMCI and AD patients, we examined the levels of proteins involved in the inflammasome pathway by Western blot analysis (Fig. 3a–h). After normalizing to the β-actin level, NLRP3, cleaved caspase-1, and mature IL-1β levels in PBMC lysates from aMCI patients were slightly higher than those in controls and significantly higher in AD patients than those in controls (Fig. 3a, b, d, h). More importantly, compared with controls, cleaved GSDMD expression increased significantly in aMCI and AD patients, indicating the aggravation of GSDMD-induced pyroptosis (Fig. 3a, f). Next, we visualized the proportion of pyroptosis in PBMCs, and the number of fluorescently stained GSDMD N-terminal positive cells in PBMCs isolated from AD patients was significantly greater than that in controls (Fig. 3i, j). These data indicate that the activation of inflammasomes can trigger the pyroptotic pathway, which supports the increase in peripheral inflammation in patients with aMCI or AD.

### IL-1β level in plasma is positively correlated with aMCI and AD progression

**Fig. 4:**
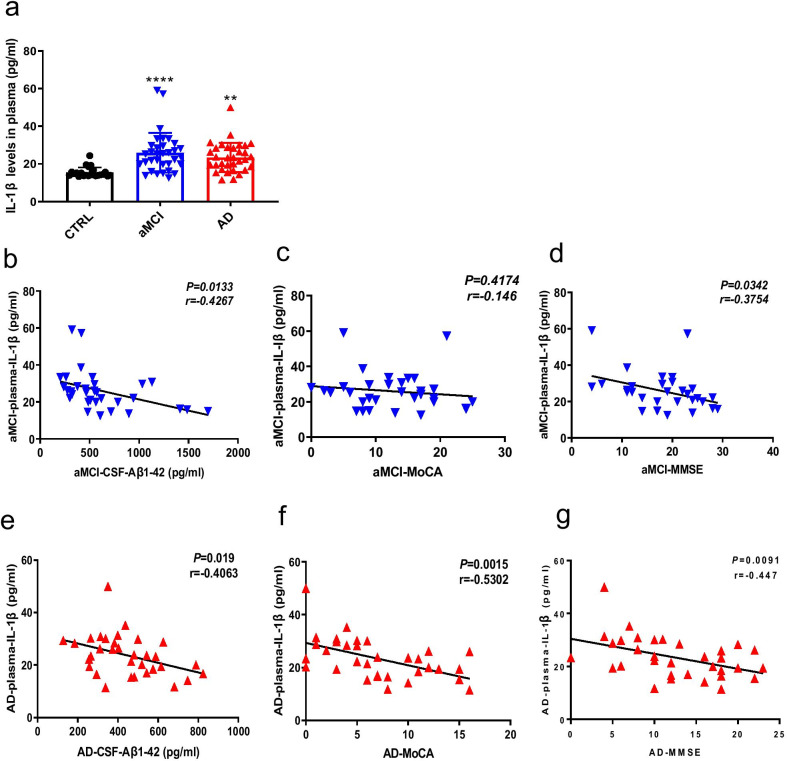
IL-1β level in plasma of aMCI and AD patients, as well as controls, and its correlation with clinical characteristics. **a** Concentration of IL-1β protein as measured by ELISA in plasma from patients with aMCI or AD, and controls. **b**–**d** Correlation of the IL-1β level in plasma and the Aβ1-42 level in CSF from patients with aMCI, and MoCA and MMSE scores. **e**–**g** Correlation of the IL-1β level in plasma and the Aβ1-42 level in CSF from patients with AD, and MoCA and MMSE scores. **P < 0.01, ****P < 0.001. *n* = 20 for control, *n* = 33 for aMCI, *n* = 33 for AD. Data are expressed as means ± SEM. One-way ANOVA for **a**. The correlation was determined by calculating correlation coefficients

Plasma specimens are relatively easy to obtain. As such, they are often used in routine analyses, thus offering the prospect of readily measuring a variety of biomarkers for many diseases. We measured the plasma IL-1β level in aMCI and AD patients, as well as controls, by ELISA. The plasma IL-1β level in patients with aMCI or AD was significantly higher than that in controls (Fig. 4a). CSF-derived Aβ1-42 is an important biomarker of AD. The Aβ1-42 level in CSF decreased, indicating that this protein accumulated in the brain parenchyma [26]. Next, we examined whether the IL-1β level in plasma correlated with the Aβ1-42 level in CSF. The results showed a negative correlation between the two biomarkers in patients with aMCI or AD (Fig. 4b, e). MMSE and MoCA scores are commonly used in clinical settings as indicators of cognitive function, with lower scores suggesting more severe cognitive impairment. We examined the correlation between the plasma IL-1β level and MMSE and MoCA scores. The plasma IL-1β level and the MMSE score were highly negatively correlated in patients with aMCI or AD (Fig. 4c, d). However, a negative correlation between the plasma IL-1β level and the MoCA score was found only in patients with AD (Fig. 4f, g).

### IL-1β level in CSF is positively correlated with aMCI and AD progression

**Fig. 5:**
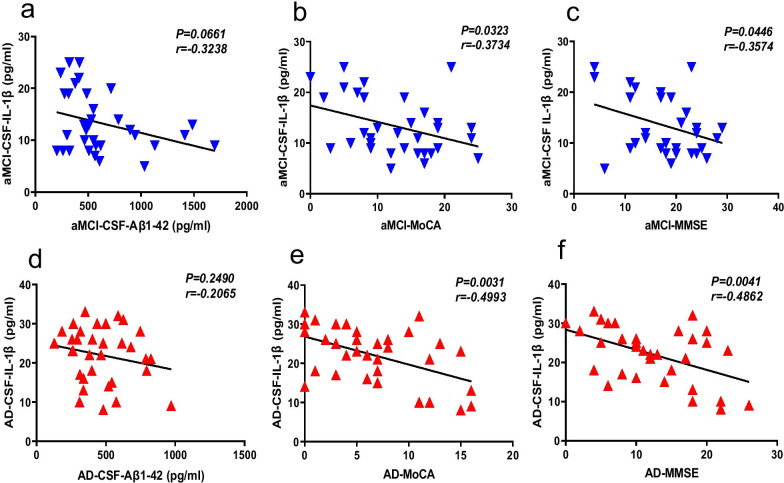
IL-1β level in CSF of aMCI and AD patients, as well as controls, and its correlation with clinical characteristics. **a**–**c** Concentration of IL-1β levels in CSF from patients with aMCI with the CSF Aβ1-42, and MoCA and MMSE scores. **d**–**f** Correlation of the IL-1β levels in CSF from patients with AD with the CSF Aβ1-42, and MoCA and MMSE scores. *n* = 33 for aMCI, *n* = 33 for AD. The correlation was established by calculating correlation coefficients

The IL-1β level in CSF was measured to determine the severity of inflammation in the brain of aMCI and AD patients. We examined the correlation between the IL-1β level in CSF and clinical characteristics, such as biomarkers and psychological evaluation scores. The results showed a weak negative correlation between the levels of IL-1β and Aβ1-42 in CSF of aMCI and AD patients (Fig. 5a, d), and a significant correlation between the IL-1β level and MMSE and MoCA scores (Fig. 5b, c, e, f), indicating that IL-1β, which associates with the progression of AD, may be a potential biomarker of neuroinflammation in AD patients.

### Positive correlation between the IL-1β level in plasma and CSF of patients with aMCI and AD

**Fig. 6:**
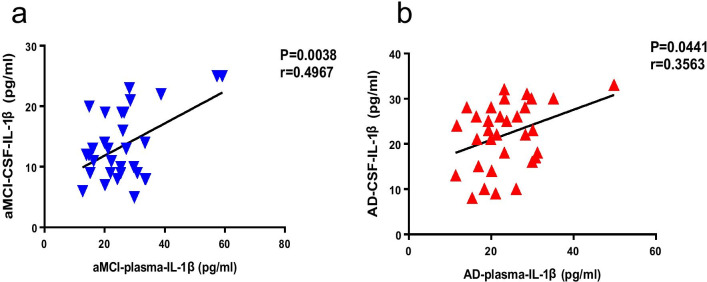
Correlation between the IL-1β level in plasma and CSF in patients with aMCI and AD. **a** Correlation of IL-1β level in plasma and CSF in patients with aMCI. **b** Correlation of IL-1β level in plasma and CSF in patients with AD. *n* = 33 for aMCI, *n* = 33 for AD. The correlation was established by calculating correlation coefficients

We examined whether the IL-1β level in CSF correlated with the IL-1β level in plasma. The IL-1β level in CSF of patients with aMCI or AD was highly positively correlated with the IL-1β level in plasma (Fig. 6). These results indicate that the degree of peripheral inflammation is strongly associated with the extent of central inflammation in patients with aMCI and AD.

### Peripheral inflammation aggravates neuroinflammation in 5 × FAD mice

**Fig. 7:**
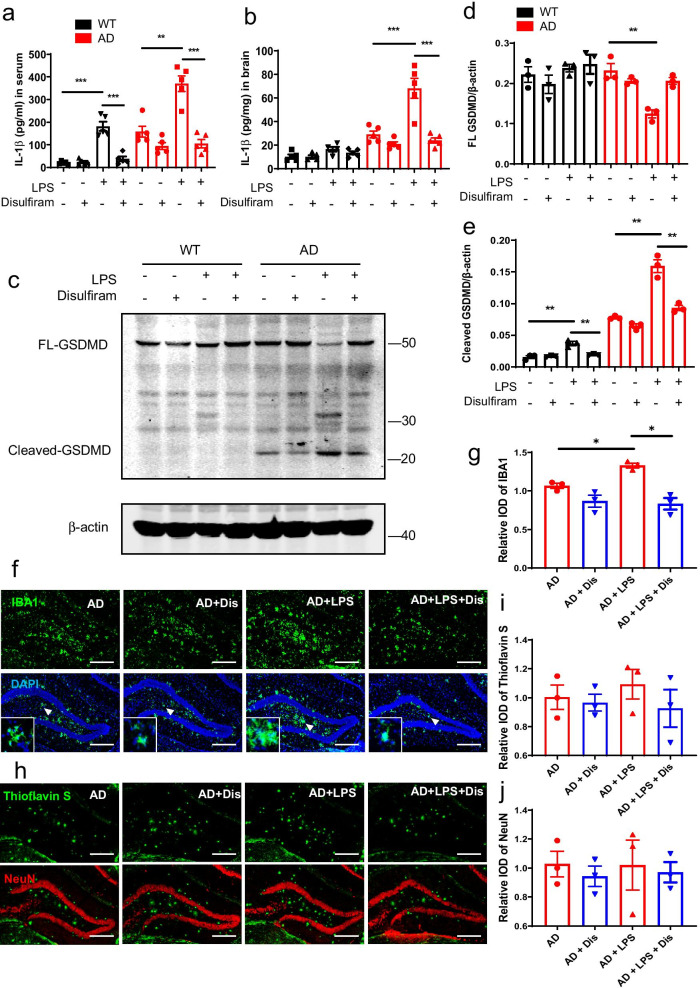
Peripheral pyroptosis affects the neuropathology of 5 × FAD mice. **a**, **b** Concentration of IL-1β protein in serum and brain specimens as determined by ELISA in the indicated mice (*n* = 5). **c** Expression and activation of GSDMD in spleen specimens as determined by Western blot analysis in the indicated mice. **d**, **e** Quantification of relative protein levels shown in **c** (*n* = 3). **f** Immunofluorescence analysis of microglia (IBA1) in hippocampus tissues from indicated mice. **g** Quantification of relative IOD values shown in **f** (*n* = 3). **h** Immunofluorescence analysis of Aβ plaques (thioflavin S) and neurons (NeuN) in hippocampus tissues from indicated mice. **i**, **j** Quantification of relative IOD values shown in **h** (*n* = 3). *P < 0.05, **P < 0.01, ****P < 0.001. Data are expressed as means ± SEM. Unpaired *t* test for **a**, **b**, **d**, **e**, **g**, and **i**, **j**

The onset of neuroinflammation in neurodegenerative diseases is not only prompted by destructive signals within the brain but also by proinflammatory cytokines released from cells of peripheral tissues. To confirm the role of pyroptosis on neuroinflammation in an in vivo AD model, we intraperitoneally injected a low dose of LPS into 5 × FAD and WT mice to induce peripheral inflammation with LPS not crossing the blood–brain barrier as previously reported [27]. The level of IL-1β in plasma and brain specimens of 5 × FAD mice was higher than that of WT mice (Fig. 7a, b). In addition, LPS increased the plasma IL-1β level in 5 × FAD and WT mice (Fig. 7a). GSDMD activation in the spleen was further up-regulated by LPS administration (Fig. 7c–e), and the level of IL-1β in brain specimens of 5 × FAD mice also increased after challenge, suggesting that peripherally activated GSDMD influences the brain in AD mice (Fig. 7b). Next, we examined the pathological features of AD after administering LPS to 5 × FAD mice. Although peripherally activated GSDMD and IL-1β did not affect the deposition of Aβ plaques and the survival of neurons around the hippocampus (Fig. 7h–j), they enhanced microglial activation in the same region of 5 × FAD mice (Fig. 7f, g), but not WT mice (Additional file 1: Fig. S1). Microglia, as innate immune system cells of the CNS, are very sensitive to disease conditions. As such, they can rapidly respond to proinflammatory cytokines. Notably, the injection of the GSDMD inhibitor disulfiram not only inhibited the activation of inflammation induced by LPS but also decreased the activation of microglia in the brain (Fig. 7a–g). Unexpectedly, disulfiram also slightly decreased the degree of peripheral and brain inflammation in 5 × FAD mice in the absence of LPS challenge (Fig. 7a–g).

## Discussion

Many studies have reported systemic inflammation to play important roles in neurodegenerative diseases, including AD [28, 29]. This study provides convincing evidence that inflammasomes and downstream pyroptosis are both activated in PBMCs of patients with aMCI or AD. RNA-seq analysis revealed that gene enrichment pathways associated with the inflammatory response were up-regulated in patients with AD. The levels of inflammasome-related genes and proteins in PBMCs of patients with aMCI or AD were greater than those in controls. In addition, the plasma IL-1β level was significantly elevated in aMCI and AD patients, and negatively correlated with the Aβ1-42 level in CSF, as well as MMSE and MoCA scores. We also observed that the IL-1β level in CSF was negatively correlated with the Aβ1-42 level in CSF, and MMSE and MoCA scores. Finally, the positive correlation between the IL-1β level in CSF and the IL-1β level in plasma was confirmed. Therefore, the IL-1β level in plasma correlated with the IL-1β level in CSF, both of which associated with AD progression. On this basis, we established an in vivo AD model to verify the effects of systemic inflammasome-induced pyroptosis on the pathophysiology of AD. Challenge with LPS enhanced peripheral inflammation and pyroptosis, whereas treatment with disulfiram decreased neuroinflammation in AD mice.

In the CNS, neuroinflammation is known to have important roles in the progression of AD [7]. Recent studies have reported that not only CSF, but the plasma IL-1β level, were significantly higher in aMCI and AD patients than those in controls [30, 31]. Systemic proinflammatory cytokines can penetrate the blood–brain barrier and increase inflammation in the brain, thereby accelerating AD progression [11]. Consistently, in our study, the plasma IL-1β level was significantly higher in patients with aMCI and AD than that in controls, and showed a positive correlation with the IL-1β level in CSF, indicating that peripherally activated IL-1β is closely associated with inflammation in the CNS. Systemic inflammasome-induced inflammation is known to contribute to a variety of neurological diseases [32–34]. The canonical NLRP3/caspase-1 inflammasome is the most studied inflammasome in AD. Studies have demonstrated that NLRP3-related gene and protein levels are elevated in Aβ-containing mice and patients with AD [35, 36]. Moreover, ASC or NLRP3 knockout decreases Aβ deposition and neurofibrillary tangle formation in transgenic AD mice [37, 38]. Here, we report the levels of inflammasome-related genes and proteins in PBMCs of patients with aMCI or AD, as well as controls. The inflammasome signaling pathway was activated in PBMCs of aMCI and AD patients. Although up-regulation of inflammatory pathways can promote aMCI and AD progression, compared to controls, we also observed an elevated IL-1β level in AD mice in the absence of LPS challenge. Studies have reported that Aβ can activate inflammasomes, indicating that inflammation may also be a consequence of AD [39, 40]. Despite the association between inflammation and AD pathophysiology, further studies are needed to completely understand how these interactions promote the onset of disease.

The downstream molecular events of inflammasome activation in pyroptosis were unclear until recently, that is, until GSDMD was identified [18]. Inflammasome-induced cleavage of the GSDMD N-terminus results in plasma membrane pore formation, eventually causing pyroptosis and IL-1β secretion [24, 25]. GSDMD can be activated by both canonical and non-canonical inflammasomes, and it has broad effects on inflammation-induced pyroptosis. We hope that this study will provide information on the association between GSDMD-induced systemic inflammation and AD, as well as guidelines for future studies. Recent studies have reported that disulfiram specifically inhibits GSDMD-induced pyroptosis [21, 41]. Our in vivo AD model showed that administration of disulfiram not only inhibited systemic inflammation and microglial activation in AD mice enhanced by LPS, but also slightly alleviated inflammation in AD mice in the absence of LPS challenge. After validating these target proteins as biomarkers for the diagnosis of AD or the assessment of disease progression, the next step will be to target these biomarkers for potentially new and improved treatments for the growing number of individuals with AD worldwide. Moreover, the relationship between inflammasome-induced pyroptosis and other important cellular processes and pathways, such as autophagy, should be explored. For instance, recent studies have demonstrated that mitophagy and chaperon-mediated autophagy can inhibit NLRP3 inflammasomes and alleviate the pathological features of AD in mice [42–44].

## Conclusions

In summary, our results provide evidence that the NLRP3/caspase-1/GSDMD signaling pathway is peripherally activated in patients with aMCI and AD. Systemic inflammasome-induced pyroptosis increases the IL-1β level and aggravates the pathophysiology of aMCI and AD.

## Supplementary Information

Below is the link to the electronic supplementary material.

**Additional file 1: Figure S1.** Glial cells in AD and WT mice treated with LPS and disulfiram. **a**, **b** Immunofluorescence analysis of astrocyte (GFAP) and microglia (IBA1) in the indicated mice, and quantification of relative IOD values. Data are expressed as means ± SEM (*n* = 3). Unpaired *t* test for **a**, **b**.

## Data Availability

The data sets and materials supporting the conclusions of this study are included within the article.

## Abbreviations

AD: Alzheimer’s disease
GSDMD: Gasdermin D
aMCI: Amnestic mild cognitive impairment
MMSE: Mini-mental State Examination
MoCA: Montreal Cognitive Assessment
PBMCs: Peripheral blood mononuclear cells
CSF: Cerebrospinal fluid
LPS: Lipopolysaccharides
Aβ: β-Amyloid
BBB: Blood–brain barrier
CNS: Central nervous system
PRR: Pattern recognition receptor
NLR: NOD-like receptor
ASC: Apoptosis-associated speck-like protein containing a CARD
AIM2: Absent-in-melanoma 2
ELISA: Enzyme-linked immunosorbent assay
FAD: Familial Alzheimer’s disease
SEM: Standard error of the mean
GSEA: Gene set enrichment analysis
