# Supplementary figures and images for: Systemic inflammasome activation and pyroptosis associate with the progression of amnestic mild cognitive impairment and Alzheimer’s disease
Source: J Neuroinflammation. 2021 Dec 2;18:280. doi: 10.1186/s12974-021-02329-2 (PMC8638109; doi:10.1186/s12974-021-02329-2)

## Slide 1
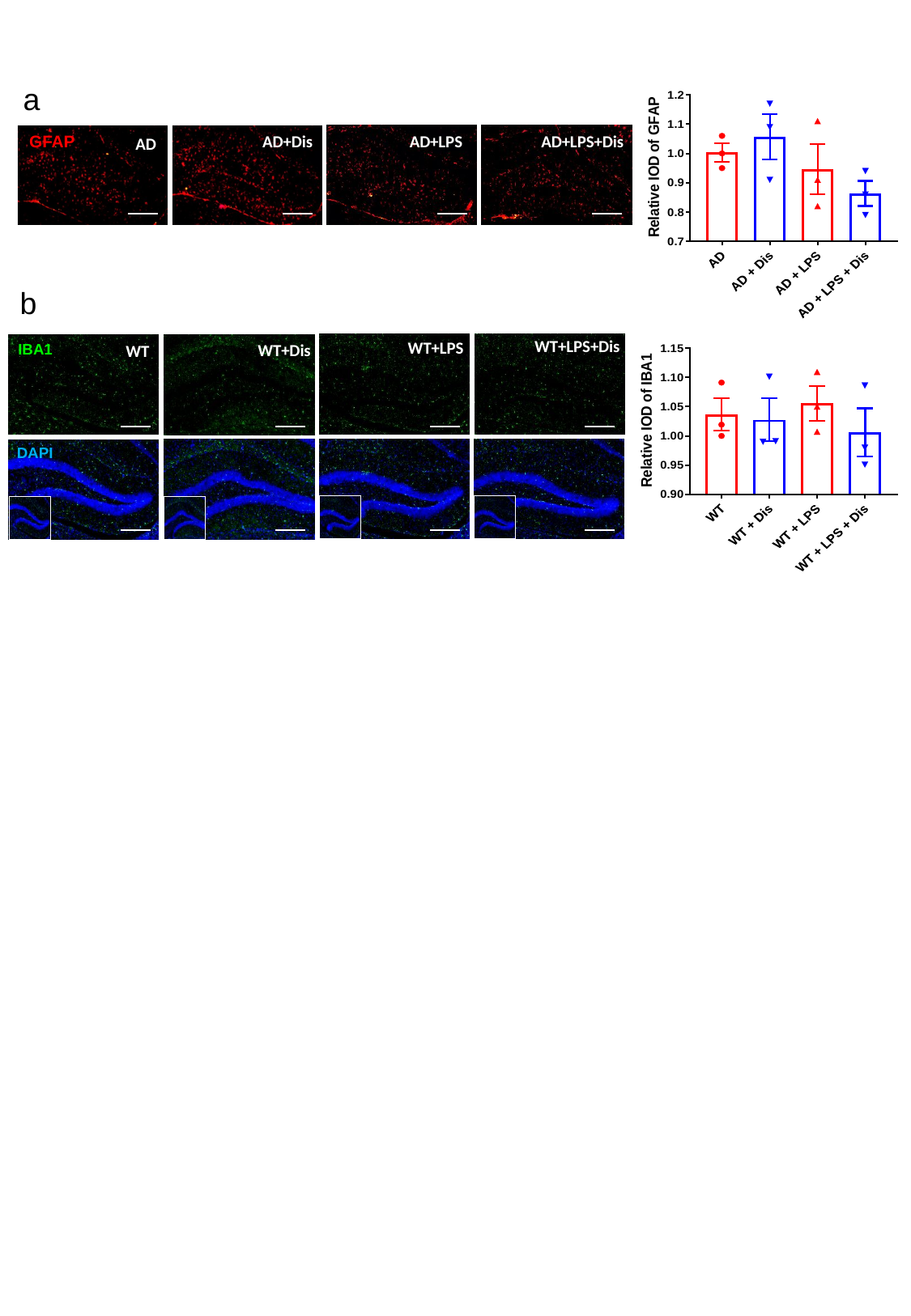

a
AD+LPS
AD+LPS+Dis
AD+Dis
GFAP
AD
b
WT+LPS+Dis
WT+LPS
IBA1
WT+Dis
WT
DAPI

Supplement: Supplementary file 1 — Additional file 1: Figure S1. Glial cells in AD and WT mice treated with LPS and disulfiram. a, b Immunofluorescence analysis of astrocyte (GFAP) and microglia (IBA1) in the indicated mice, and quantification of relative IOD values. Data are expressed as means ± SEM (n = 3). Unpaired t test for a, b. [file 12974_2021_2329_MOESM1_ESM.pptx]
